# Progression from High Insulin Resistance to Type 2 Diabetes Does Not Entail Additional Visceral Adipose Tissue Inflammation

**DOI:** 10.1371/journal.pone.0048155

**Published:** 2012-10-24

**Authors:** Nuria Barbarroja, Chary Lopez-Pedrera, Lourdes Garrido-Sanchez, Maria Dolores Mayas, Wilfredo Oliva-Olivera, Maria Rosa Bernal-Lopez, Rajaa El Bekay, Francisco Jose Tinahones

**Affiliations:** 1 Servicio de Endocrinología y Nutrición, Hospital Virgen de la Victoria (Fundación IMABIS), Málaga, Spain; and CIBER Fisiopatología de la Obesidad y Nutrición CB06/03, Instituto de Salud Carlos III, Madrid, Spain; 2 Unidad de Investigación, Hospital Reina Sofía-IMIBIC, Córdoba, Spain; 3 CIBERDEM, Hospital Universitari de Tarragona Joan XXIII, IISPV, Universitat Rovira i Virgili, Tarragona, Spain; 4 Área de Fisiología, Universidad de Jaén, Jaén, Spain; University of Tor Vergata, Italy

## Abstract

Obesity is associated with a low-grade chronic inflammation state. As a consequence, adipose tissue expresses pro-inflammatory cytokines that propagate inflammatory responses systemically elsewhere, promoting whole-body insulin resistance and consequential islet β-cell exhaustation. Thus, insulin resistance is considered the early stage of type 2 diabetes. However, there is evidence of obese individuals that never develop diabetes indicating that the mechanisms governing the association between the increase of inflammatory factors and type 2 diabetes are much more complex and deserve further investigation. We studied for the first time the differences in insulin signalling and inflammatory pathways in blood and visceral adipose tissue (VAT) of 20 lean healthy donors and 40 equal morbidly obese (MO) patients classified in high insulin resistance (high IR) degree and diabetes state. We studied the changes in proinflammatory markers and lipid content from serum; macrophage infiltration, mRNA expression of inflammatory cytokines and transcription factors, activation of kinases involved in inflammation and expression of insulin signalling molecules in VAT. VAT comparison of these experimental groups revealed that type 2 diabetic-MO subjects exhibit the same pro-inflammatory profile than the high IR-MO patients, characterized by elevated levels of IL-1β, IL-6, TNFα, JNK1/2, ERK1/2, STAT3 and NFκB. Our work rules out the assumption that the inflammation should be increased in obese people with type 2 diabetes compared to high IR obese. These findings indicate that some mechanisms, other than systemic and VAT inflammation must be involved in the development of type 2 diabetes in obesity.

## Introduction

Over the last few years, the number of people with diabetes mellitus has massively increased, becoming one of the most important public health challenges globally. On the onset of type 2 diabetes mellitus, obesity is the major cause. Recently, major advances have been made in understanding the mechanisms that are involved in the pathogenesis of T2D. However, to date the only effective therapy used in the treatment of this disorder is the weight loss and the lately accepted bariatric surgery [1]. Thus, a critical challenge is to recognize who among the obese subjects will be at high risk of developing T2D eventually.

It is noteworthy that many of the insulin resistant individuals do not become diabetic, because their β -cells are somehow able to cope with the elevated insulin request. In fact, only one-third of insulin resistant obese individuals develop type 2 diabetes mellitus. The precise mechanisms that lead to the β-cell dysfunction are incompletely understood, although some processes have been postulated such as oxidative stress, endoplasmic reticulum stress, lipotoxicity and increased levels of inflammation [2], [3], [4]. Interestingly, all these factors may elicit an inflammatory response, whereas some may be the result of the inflammation [5], [6].

Speaking about inflammation in terms of type 2 diabetes associated with obesity, adipose tissue plays an important role as a pathogenic site of obesity-induced insulin resistance. However, all depots are not equal regarding their potential role in insulin resistance, being visceral more pathogenic than subcutaneous adipose tissue [7]. Thus, large adipocytes produce high levels of chemoattractants, promoting macrophage infiltration in adipose tissue [8]. These recluted macrophages are activated by several mechanism including free fatty acids (FFA) spilled by adipocytes, changing its state, from one that is non inflammatory to a proinflamatory state, which implies the release of a significant proportion of proinflammatory cytokines such as TNFα, IL-1β and IL-6 [9]. These molecules are disseminated to the circulation and affect others distant organs, including pancreas, liver, skeletal and cardiac muscle. How is insulin resistance induced at intracellular level? The mechanisms involved have been widely studied.

Kinases such as Jun N-terminal kinase (JNK), IKKβ and the nuclear transcription κB (NF-κB) are activated by elevated levels of TNFα, IL-1β and IL-6 through classical receptor-mediated mechanisms. The activation of theses kinases increase the expression of many markers and potential mediators of inflammation that can cause insulin resistance. Under these conditions, insulin receptor substrate 1 (IRS-1) becomes one of the main targets for these kinases, inducing its phosphorylation at serine sites that negatively regulate normal signaling through the insulin receptor/IRS-1 axis and produce impaired insulin action [10], [11], [12].

Other kinases such as extracellular signal- regulated kinase (ERK) 1/2 and STAT3 have been also demonstrated to be involved in obesity [13], [14].

Thus, chronic low-grade inflammation has been postulated as one of the key steps in the pathogenesis of obesity-induced T2D. Many recent studies focused on adipose tissue have shown an increase expression of inflammatory molecules in type 2 diabetes associated with obesity. However, most of these studies have been conducted in a group of obese subjects that either were insulin resistance or type 2 diabetic or a mixture of both and were further taking antiadiabetic or other drugs.

Being clear that inflammation related to obesity contributes to insulin resistance, the first step in the development of T2D, we set out whether there is a difference in the state of inflammation among insulin resistant subjects and diabetic patients. Thus, we hypothesised that analyzing these two specific groups (classified based on the insulin resistance and diabetes state with no treatment given that could alter the results) could provide some important clues on the mechanisms linking insulin resistance, inflammation and diabetes.

Here, we analyzed the differences in insulin signalling and inflammatory pathways in the blood and visceral adipose tissue of equally obese people that just differ in their insulin resistance and diabetes state. Specifically we evaluated: a) the changes in proinflammatory markers and lipid content from serum; b) the variations in macrophage infiltration and polarization, mRNA expression of pro and anti-inflammatory cytokines and transcription factors (TNFα, IL-1β, IL-6, IL-10 and NF-κB, IκBα, JNK, STAT3); c) the status of activation of kinases involved in inflammation and insulin signalling (JNK, ERK, STAT3, IRS1, IRS2 and NF- κB) in visceral adipose tissue.

## Results

### Anthropometric and Biochemical Variables of the Healthy Leans and Morbidly Obese Patients

The anthropometric and biochemical variables of the morbidly obese patients with high insulin resistance and diabetes and the lean healthy controls are summarized in Table 1.

**Table 1:** Baseline biological characteristics of the healthy subjects and morbidly obese patients.

	Leans	High IR-MO	T2D-MO
Male/female (n/n)	10/10	10/10	10/10
Age (years)	40.85±2.76^a^	37.38±2.17^a^	41.78±1.73^a^
Weight (Kg)	66.38±2.60^a^	158.12±5.39^b^	150.37±4.25^b^
BMI (Kg/m^2^)	22.81±0.53^a^	55.76±1.61^b^	54.44±0.92^b^
Waist circumference (cm)	80.83±2.22^a^	148.16±4.43^b^	144.63±3.96^b^
Hip circumference (cm)	94.59±2.26^a^	156.83±3.09^b^	153.63±2.09^b^
Waist to Hip ratio	0.85±0.02^a^	0.94±0.02^b^	0.93±0.02^b^
Serum insulin (UI/ml)	7.37±0.58^a^	35.32±2.00^b^	29.30±2.12^c^
HOMA-IR	1.48±0.11^a^	8.79±0.47^b^	9.72±1.33^b^
Serum glucose, mg/dl	80.61±2.43^a^	101.57±2.38^b^	141.40±7.74^c^
Serum cholesterol, mg/dl	198.09±8.68^a^	188.73±7.38^a^	205.50±6.39^a^
HDL cholesterol, mg/dl	57.09±3.23^a^	41.05±3.08^b^	42.60±2.14^b^
LDL cholesterol, mg/dl	118.28±6.35^a^	120.74±7.70^a^	128.74±5.54^a^
Triglycerides, mg/dl	88.90±9.66^a^	160.84±21.51^b^	157.94±19.13^b^
GOT (units/l)	20.71±1.33^a^	21.83±1.52^a^	26.00±3.60^a^
GPT (units/l)	38.80±2.16^a^	47.66±3.71^a,b^	52.75±5.21^b^
GGT (units/l)	29.33±3.10^a^	40.63±7.63^a,b^	64.75±13.33^b^
Leptin (ng/ml)	7.74±1.78^a^	71.14±7.25^b^	78.37±8.03^b^
Adiponectin (ng/ml)	25.47±4.08^a^	5.58±0.63^b^	5.02±0.63^b^
FFA (mmol/l)	0.50±0.05^a^	0.57±0.04^a^	0.61±0.06^a^
**Uric acid**	4.18±0.25^a^	6.46±0.26^b^	6.18±0.35^b^

1 Values are means ± SEM, n = 12. HDL-c = High density lipoprotein-cholesterol; LDL-c = Low density lipoprotein-cholesterol; GPT = Glutamic pyruvate transaminase; GGT = Gamma glutamil transferase; GOT = Glutamic oxalacetic transaminase. Significant differences (Duncan; p<0.05) are indicated with different words.

Plasma fasting insulin, glucose levels and HOMA values were seen to be significantly higher in the obese population compared to the lean group. As expected, a decrease of serum insulin levels and an increase of serum glucose levels were observed in T2D-MO patients compared to high IR-MO subjects.

Regarding lipid profile, triglycerides levels were found significantly elevated in the obese population versus control group, whereas a significant decrease of HDL cholesterol levels was seen in all obese subjects compared with lean individuals. In contrast, no differences were observed in total cholesterol, LDL cholesterol and free fatty acids levels between the three groups, lean and two obese subgroups. Insulin resistant individuals and diabetic obese showed no significant differences in all of these lipid parameters.

When we compared the clinical parameters between men and women, we detected significant differences in the levels of HDL (men 41.33±2.61 and women 52.54±13.68, p = 0.003) and GPT (glutamic pyruvate transaminase) (men 54.96±3.74 and women 39.74±2.26, p = 0.001) between men and women. These sex-related differences have already been reported, pointing sex steroid hormones as the responsible for this alteration [15], [16].

### Inflammation Markers in Blood and Visceral Adipose Tissue from Morbidly Obese with High Insulin Resistance and Diabetes

**Figure 1:**
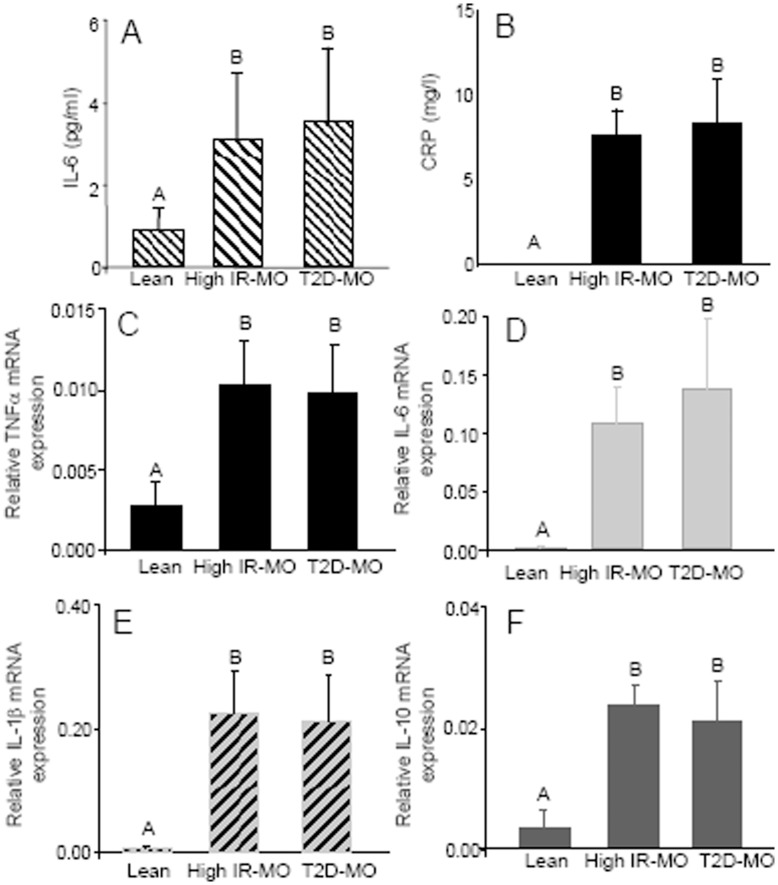
Increased levels of proinflammatory cytokines in serum and visceral adipose tissue from high IR-MO and T2D-MO subjects. (**A and B**) IL-6 and CRP plasma levels were increased in blood from the morbidly obese group compared with the undetectable amounts observed in healthy lean controls (p<0.01). There were not significant differences in the levels of these markers between high insulin resistance and diabetic groups. (**C, D, E and F**) There was a great increase of proinflammatory cytokines mRNA expression (TNFα, IL-1β, IL-6 and IL-10) in VAT of MO patients compared with controls (p<0.01). Of note, elevated levels of TNFα, IL-1β, IL-6 and IL-10 mRNA in MO with high insulin resistance degree were not statistically different from the levels found in T2D-MO. Significant differences (Duncan; p<0.05) are indicated with different words.

In agreement with the established evidence, we found increased levels of IL-6 and protein C reactive (CRP) in blood from the morbidly obese group compared with the undetectable amounts of these proinflammatory markers observed in healthy lean controls (p<0.01), a feature that characterizes obesity. However, there was not significant difference in the levels of these markers when the obese group was classified based on the degree of insulin resistance and diabetes state (Figure 1A and B).

As expected, we observed a great increase of proinflammatory cytokines (TNFα, IL-1β and IL-6) expression in visceral adipose tissue of morbidly obese patients compared with the one existing in lean individuals (p<0.01). Moreover, as previously reported, we also found high levels of anti-inflammatory IL-10 in VAT of morbidly obese patients compared with lean (p<0.01) [17]. Of note, elevated levels of TNFα, IL-1β, IL-6 and IL-10 mRNA in MO with high insulin resistance degree were not statistically different from the levels found in T2D-MO (Figure 1C, D, E and F).

**Figure 2:**
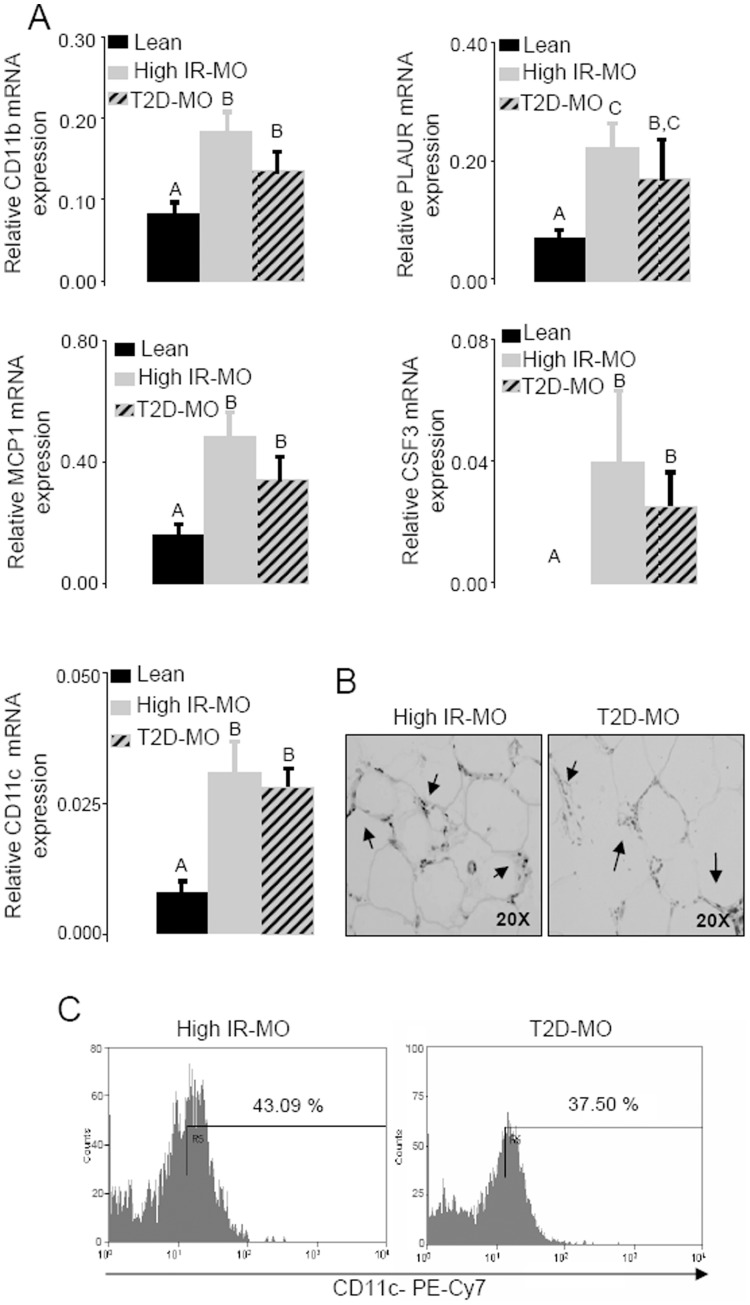
Elevation of infiltrated macrophages in visceral adipose tissue from high IR-MO and T2D-MO subjects. (A) The mRNA expression of macrophage markers CD11b, PLAUR, MCP1, CSF3 and CD11c was increased in the MO group compared to healthy leans (Duncan; p<0.05). More importantly, the expression of macrophage markers was not increased in the visceral adipose tissue of T2D-MO compared to high IR-MO patients. (B) Immunohistochemical detection of CD68+ macrophages in VAT from both, high IR-MO and T2D-MO patients, characteristically showed macrophages surrounding adipocytes forming the typical crowns. No changes were detected between both morbidly obese groups. Significant differences (Duncan; p<0.05) are indicated with different words. (C) CD11c detection in ATMs through flow cytometry indicated that there was no significance difference in CD11c protein expression in high IR-MO and T2D-MO subjects.

Given the fact that macrophage infiltration has been recognized as an important determinant factor of the metabolic complications associated with obesity, we analyzed the presence of macrophages in visceral adipose tissue in lean, high IR-MO and T2D-MO groups. PCR real time analysis showed that the expression of macrophage markers cluster of differentiation molecule 11B (CD11b), plasminogen activator urokinase receptor (PLAUR), monocyte chemotactic protein 1 (MCP1) and colony-stimulating factor 3 (CSF3) was increased in the morbidly obese group compared to healthy leans. More importantly, our data also showed that the expression of macrophage markers was not increased in the adipose tissue of T2D-MO compared to high IR-MO patients (Figure 2A).

Further analysis of the presence of macrophages was obtained using immunoreactivity for the CD68 marker. As shown in the figure 2B, VAT from both, high IR-MO and T2D-MO patients, characteristically showed macrophages surrounding adipocytes forming the typical crowns. In agreement with PCR data, no changes were detected between both morbidly obese groups.

Regarding the phenotype of these macrophages, real time PCR analysis of CD11c (a specific marker for M1 macrophage subtype) revealed a higher expression in the obese population compared to lean controls. More importantly, there was no difference in the elevated CD11c mRNA levels between both obese subgroups (Figure 2A). Moreover, flow cytometry analysis carried out on isolated macrophages from visceral adipose tissue (ATMs) showed that CD11c protein expression was similar in both groups (Figure 2C) (high IR-MO 48.5±11.5% and T2D-MO 45.8±8.6%).

Correlation studies were performed between mRNA expression of cytokines and macrophage markers in VAT from morbidly obese patients. Increased mRNA expression of TNFα, IL-1β and IL-6 showed a significant positive correlation with CD11b, CSF3, PLAUR, MCP1 and CD11c, indicating that proinflammatory cytokines in VAT are mainly produced by macrophages (Table 2).

**Table 2:** Correlations between mRNA expression of cytokines and macrophage markers in visceral adipose tissue of morbidly obese patients.

	IL-β	IL-6	TNFα
**CD11b**	0.119	0.052	0.455^a^
**CSF-3**	0.596^a^	0.635^a^	0.050
**PLAUR**	0.708^a^	0.763^a^	0.118
**MCP-1**	0.448^a^	0.510^a^	0.365^a^
**CD11c**	0.630^a^	0.580^a^	0.200

Correlations were determined by Spearman’s correlation coefficient test.

a p<0.01;

b p<0.05 CD11b, Cluster of differentiation molecule 11B; PLAUR, Plasminogen activator urokinase receptor, MCP-1; Monocyte chemotactic protein-1; CSF-3, Colony-stimulating factor 3; CD11c, Integrin, alpha X; IL-1β, interleukin 1β; IL-6, Interleukin 6; TNFα, Tumor necrosis factor α.

### Activation of Inflammatory Intracellular Pathways: JNK1/2, ERK1/2, STAT3 and NF-κB

**Figure 3:**
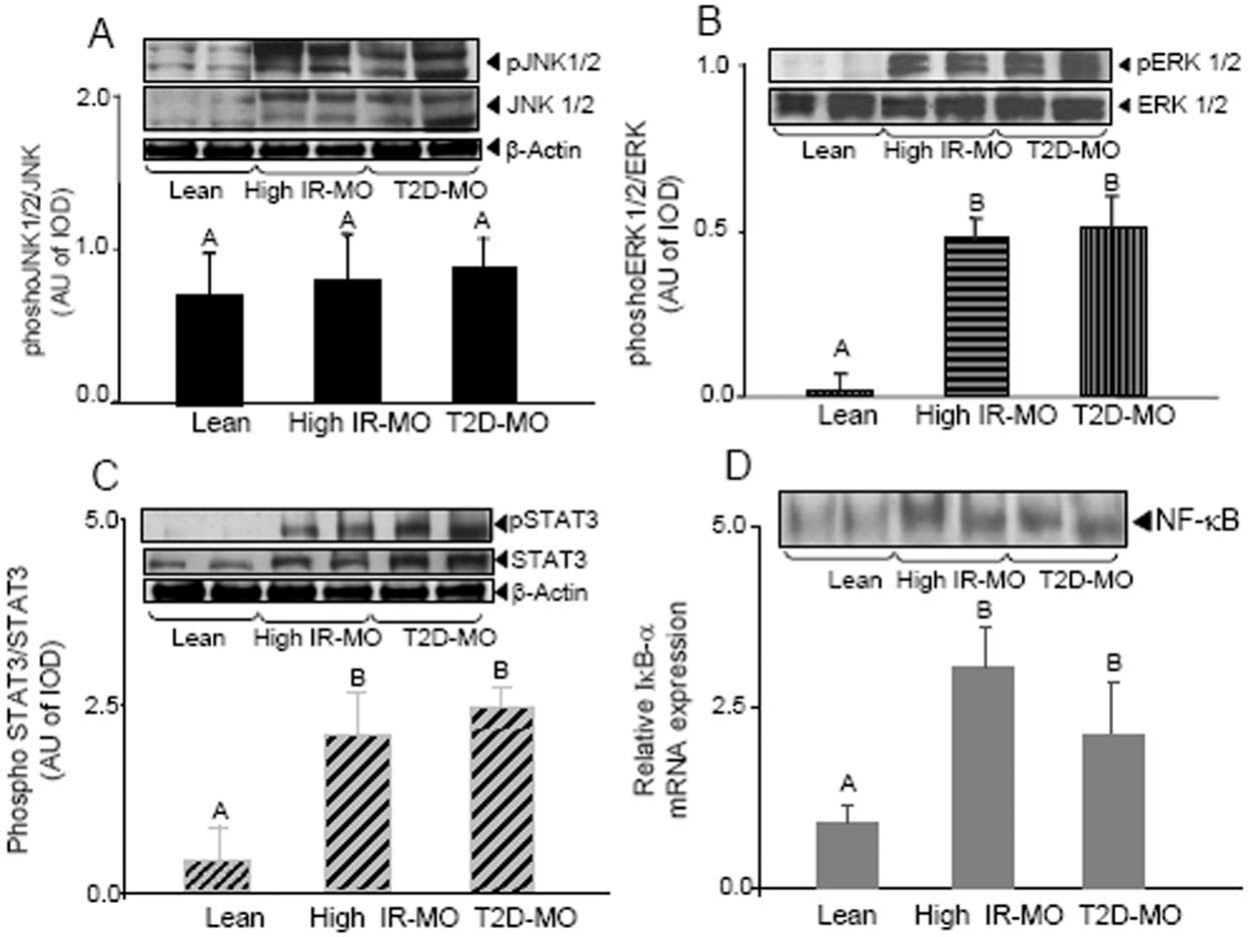
Intracellular inflammatory pathways in visceral adipose tissue from non obese, high IR-MO and T2D-MO individuals. (**A**) Expression and phosphorylation of JNK 1/2 isoforms: All obese patients, independently of high degree of insulin resistance and diabetes state, had significantly elevated levels of JNK 1 and 2 expression and phosphorylation versus lean group. (**B**) Expression and activation of ERK1/2 isoforms: All samples had similar levels of constitutive ERK 1/2 expression. VAT of lean subjects showed an undetectable ERK1/2 activation, in contrast to the high levels of ERK 1/2 activation seen in morbid obese patients (high IR-MO and T2D-MO). No significant differences were observed between both obese groups. (**C**) Expression and phosphorylation of STAT3: Both, phosphorylation and expression of STAT3 were significantly increased in VAT from MO individuals versus lean controls. Although there were not differences in the observed phosphorylation of STAT3 between high IR-MO and diabetic groups, elevated levels of expression were seen in diabetic individuals comparing with high IR-MO. (**D**) NF-κB pathway: There was a significant increase of IκB-α mRNA expression in VAT of MO compared to non obese subjects. Moreover, there were no significant differences in the high levels observed in both morbidly obese groups. EMSA technique revealed that NF-κB activation was significantly elevated in VAT of MO patients compared to the low levels seen in lean individuals. NF-κB activity levels were similar when the MO group was divided in high IR and T2D subgroup. Significant differences (Duncan; p<0.05) are indicated with different words.

After having observed equal elevated levels of cytokines and macrophages markers in visceral adipose tissue from high IR-MO and T2D-MO patients, we then set to investigate the activation of intracellular signalling involved in inflammation. JNK plays a pivotal role in metabolic conditions such as obesity, insulin resistance, and type 2 diabetes [18]. Western blot analysis showed that all obese patients, independently of high degree of insulin resistance and diabetes state, had significantly elevated levels of JNK 1 and 2 expression and phosphorylation versus lean group. Of note we did not find differences between high IR-MO and T2D-MO groups (Figure 3A). Thus, elevated phosphorylation of JNK1/2 observed in all MO group was probably due to the increase of its expression.

Another relevant kinase involved in inflammation is ERK, identified as a regulatory node controlling the inflammation signalling network. Thus, we next analyzed the basal expression and activation of ERK 1/2 in VAT of lean healthy subjects and our two experimental obese groups. All samples had similar levels of constitutive ERK 1/2 expression. VAT of lean subjects showed an undetectable ERK1/2 activation, in contrast to the high levels of ERK 1/2 activation seen in morbid obese patients (high IR-MO and T2D-MO) compared with controls. No significant differences were observed between both obese groups (Figure 3B).

It has been shown that IL-6 directly activates the signal transducer and activator of transcription 3 (STAT3), which in turn modulate CRP gene transcription. Moreover, adiponectin was shown to downregulate the levels of STAT3 [19]. These facts strongly link STAT3 protein with obesity and insulin resistance. In our three experimental groups, both phosphorylation and expression of STAT3 were found significantly increased in VAT from morbidly obese individuals versus lean subjects (Figure 3C). Although there were not differences in the observed phosphorylation of STAT3 between high IR-MO and diabetic groups, elevated levels of expression were seen in diabetic individuals comparing with high IR-MO.

Thereafter we studied the activation of the NF-κB signalling pathway. For this purpose, we analyzed the mRNA expression of p65 subunit (Rel-A) and the NF-κB inhibitor, IκB-α and NF-κB (p65/p50) DNA binding. Real time PCR data showed no significant changes in mRNA expression levels of p65 subunit in VAT of our three experimental groups (lean, high IR-MO and T2D-MO) (data no shown). However, we did observe a significant increase of IκB-α mRNA expression in VAT of morbid obese compared to non obese subjects. Moreover, there were no significant differences in the high levels observed in both morbidly obese groups (Figure 3D). Using EMSA technique to analyze the DNA binding of the transcriptionally active NF-κB (p65/p50), we found that NF-κB activation was significantly elevated in VAT of morbidly obese patients compared to the low levels seen in lean individuals. Of note, the values of NF-κB activity were similar when the morbidly obese group was divided in high IR and T2D subgroup (Figure 3D).

As suggested by the Spearman’s correlation, altered intracellular signalling and levels of proinflammatory cytokines seem to be coordinate in VAT of morbidly obese regardless of the insulin resistance and diabetes state (Table 3). There was a strong correlation between mRNA expression levels of IL-1α and IL-6 which in turn correlated with NF-κB signaling (p65 and IκB-α mRNA). In addition, elevated TNFα mRNA expression levels correlated with mRNA expression of JNK, which in turn correlated with the expression of p65 and IκB-α. That data suggest that inflammatory pathways activated by interleukins might be different of those elicited by TNFα, although both of them converge eventually on the activation of NF-κB. All correlations coefficients (Rs) and probability values for these correlations are stated in the table 3.

**Table 3:** Correlations between mRNA expression of cytokines and inflammatory modulators in visceral adipose tissue of morbidly obese patients.

	IL-1β	IL-6	TNFα	JNK	p65	IκB
**IL-1β**	1.000					
**IL-6**	0.630^a^	1.000				
**TNFα**	0.042	0.008	1.000			
**JNK**	0.095	0.025	0.690^a^	1.000		
**p65**	0.248^b^	0.236^b^	0.576^a^	0.731^a^	1.000	
**IκB**	0.492^a^	0.737^a^	0.120	0.560^b^	0.572^b^	1.000

Correlations were determined by Spearman’s correlation coefficient test.

a p<0.01;

b p<0.05 IL-1βb, interleukin 1β; IL-6, Interleukin 6; TNFα, Tumor necrosis factor α;JNK, Jun kinase; IκB, Inhibitor kinaseB.

### Insulin Pathway Modulation

**Figure 4:**
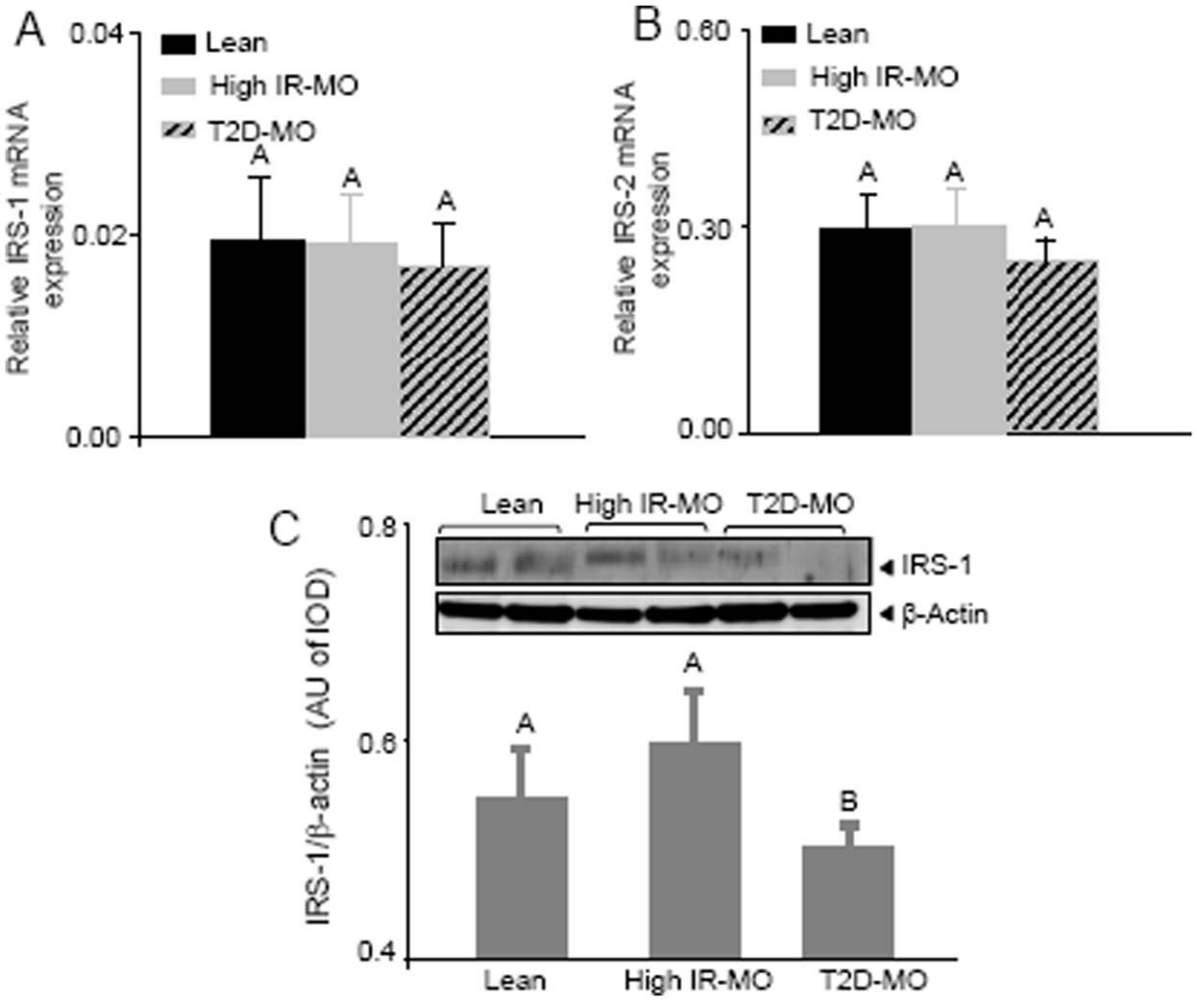
IRS-1 and IRS-2 expression in visceral adipose tissue from non obese, high IR-MO and T2D-MO subjects. (**A and B**) mRNA expression levels of IRS-1 and IRS-2: There were no changes in the mRNA expression of these genes between the lean and the morbidly obese groups. (**C**) IRS-1 protein levels were significantly decreased in VAT of T2D-MO subjects compared to high IR-MO and lean controls (Duncan; p<0.05). Significant differences (Duncan; p<0.05) are indicated with different words.

It is well known that both insulin receptor substrate (IRS) 1 and 2 are key regulators in the signaling of insulin and its function is susceptible of being blocked under insulin resistance conditions [20], [21]. Thus, we analyzed the mRNA expression of both IRS-1 and IRS-2 in VAT from our three experimental groups. Surpringsily, real time PCR analysis showed no changes in the expression of these genes in the lean and the morbidly obese groups (Figure 4A and B). However, IRS-1 gene expression data was not associated with IRS-1 protein levels, which were found significantly decreased in VAT of T2D-MO subjects compared to high insulin resistant morbidly obese and lean controls (Figure 4C).

## Discussion

There is evidence that some obese people can go their entire lives without ever developing diabetes. Indeed, it has been suggested that inflammation-associated obesity increase according to the worsening of the disease. In the present work, we deepened in the study of the mechanisms involved in the onset of type 2 diabetes mellitus in obesity by analyzing for the first time, serum lipid content, inflammation markers, macrophage infiltration and intracellular pathways in VAT from a cohort of extremely well weight/metabolic state matched obese distributed into two experimental groups, high insulin resistant degree (prediabetes phase) and diabetes state. We here provide evidence that there are inflammatory markers that are related to insulin resistance but these cannot predict the development of T2D indicating that T2D is not simply related to the inflammation degree, supporting the fact that others mechanisms must be implicated in predisposing individuals to develop this disease.

The direct comparison between our equally obese individuals also provided interesting data about glucolipoxicity. Obese people were characterized by an increase of triglycerides levels and decrease of HDL cholesterol values. No changes were seen in total cholesterol, LDL cholesterol and FFA levels in obese group compared to controls. Similar data have been shown in a recent study carried out in a group of 40 obese women [22]. Of note, our data showed that this deregulated lipid profile observed in obese patients had no statistically significant differences between high IR-MO and T2D-MO subjects. It has recently been suggested that elevated levels of glucose and FFA synergize to induce pancreaticβ-cell death [23], [24]. In this regard, serum glucose levels were found increased in T2D-MO compared to high IR-MO, probably due to the impaired β-cell function; however any changes were observed in FFA levels.

In searching for discriminative markers related to inflammation, two commonly studied inflammatory markers in plasma, IL-6 and CRP, were evaluated. We have previously provided evidence that proinflamatory cytokines such as IL-1β, IL-6 and TNFα are increased in blood and adipose tissue in obese patients with high insulin resistance compared to low insulin resistant obese subjects, demonstrating the strong link between inflammation and insulin resistance development [25]. As insulin resistance is considered a prediabetes stage and inflammation has been linked to the onset of this process, it might be expected to find higher levels of inflammation markers in obese patients that have developed T2D versus those obese people that keep an insulin resistant state. However, our data dismissed this belief, demonstrating that levels of IL-6 and CRP in blood were not significantly different between high IR-MO and T2D-MO patients. By contrast, a recent study conducted in similar subgroups of obese patients (high insulin resistant and T2D obese) showed an increase of IL-6 plasma levels in T2D obese in comparison with high IR obese patients, although they found no differences in TNFα levels in blood from both groups of participants. However, the two cohorts are not fully comparable, since the patients included in our study were not taking any antidiabetic treatment, whereas the patients from Tordjman et al, were treated with metformin or insulin or hypolipemic drugs [26].

Our study is particularly focused on the visceral adipose tissue. Many studies centered in adipose tissue biology and the metabolic syndrome tends to focus on the subcutaneous depot given its relatively easy accessibility. Studies done in paired biopsies from subcutaneous and visceral adipose tissue have shown specific adipose tissue pattern expression of inflammatory molecules and adipokines [27], [28]. However, accumulating evidence suggests that the visceral depot is the main contributor to the pathogenesis of obesity-associated metabolic diseases [29], [30].

Thus, a great upregulation of inflammatory cytokines (including IL-6, IL-1β, TNFα and IL-10) was found in both obese populations, high IR-MO and T2D-MO, compared to lean controls, but no significant differences were observed between both obese subgroups.

Our study also provides interesting data about the presence of resident macrophages in visceral adipose tissue. It has been shown that macrophage infiltration degree is distinct in the different adipose tissue depots, being higher in visceral depot than in subcutaneous adipose tissue and may have a critical role in the pathophysiology of the cormobidities related to visceral adiposity [31]. Molecules known as macrophages markers, such as CSF-3, MCP-1 and PLAUR have been found elevated in subcutaneous adipose tissue of morbidly obese patients compared to healthy lean subjects [32]. In line with these studies we found higher expression of macrophages markers, including CD11b, PLAUR, MCP1 and CSF3 in visceral adipose tissue of our non-healthy morbidly obese patients than in lean controls. Of note, macrophage infiltration degree in T2D-MO remained at levels comparables to those of high IR-MO. We have previously demonstrated that lipid overload-associated obesity lead to changes in lipid composition which are related to adipose tissue macrophage polarization from M2 to M1 state [33]. These M1 macrophages are associated with insulin resistance and are characterized by the expression of markers such as IL-1β, TNFα and CD11c. In this sense we found no differences in the high expression level of CD11c mRNA in VAT between high IR-MO and T2D-MO patients. Moreover, similar CD11c protein expression was observed in isolated macrophages from VAT of our two experimental obese groups, suggesting that the state of macrophage polarization is similar in both. Moreover, there was a strong correlation between the expression of proinflammatory cytokines (IL-1β, IL-6 and TNFα) and M1 macrophages markers (MCP-1 and CD11c); supporting the concept these cytokines are mainly expressed by M1 macrophages in visceral adipose tissue.

Fat overload is associated with impairments in insulin signaling. This failure in insulin pathway is mediated by the cooperation of multiple stress kinases which are activated by proinflammatory cytokines, phosphorylating serine residues on the insulin receptor (IR) and IRS molecules, which in turn inhibit insulin signaling [34], [35].

The most well known kinase associated with the insulin signaling blocking is JNK. JNK1/2 adipose tissue knockout mouse model showed a reduction in weight gain, fat mass and size of adipocytes, demonstrating the important role of JNK adipose tissue in the metabolism of the whole body [36]. We have previously analyzed the expression levels of both TNFα and JNK in VAT of two morbidly obese groups divided in low IR and high IR, finding that levels of both TNFα and JNK1/2 did not allow the discrimination between low IR and high IR individuals [25]. Here, we provide evidence that adding one step more in obesity complications such as T2D-MO group, TNFα and JNK expression did not experiment any significant increase in comparison with the previous stage, high insulin resistance.

Obesity is also associated with an increase in NF-κB DNA-binding activity and activation of ERK [37], [38]. Proinflammatory cytokines are able to activate these pathways. Thus, in response to the elevated levels of cytokines observed in both obese groups, we indicated that these intracellular pathways were equally activated in VAT of T2D-MO and high IR-MO patients, providing more evidence that intracellular inflammatory signals activation levels are no significant different between both experimental obese subgroups.

In the adipose tissue context, leptin exerts its actions through activation of JAK/STAT signaling [39]. Thus, the relevant role of STAT3 in the effect of leptin in food intake has recently been demonstrated [40]. Although many studies have focused on the role of this kinase in the effects of leptin, none has presented any data about expression and activation levels in visceral adipose tissue in morbidly obesity. In this sense, higher levels of STAT3 expression were found in VAT of obese people than in lean controls, which could have been expected according to the high levels of leptin observed in plasma of obese people. Despite the increased expression of STAT3 observed in T2D-MO in comparison with high IR-MO subjects, western blot data showed no differences in the high phosphorylation of STAT3 between both obese groups. Thus, leptin-STAT3 signal did no allow us to discriminate between T2D and high IR morbidly obese patients.

It has been well described that IRS1 and IRS2 are target of inflammation modulators, causing the block of the insulin pathway [18], [19]. In the present study, we also evaluated the effect of the altered proinflammatory profile (observed in blood and visceral adipose tissue) in the insulin signaling of our obese population. At VAT level, our results revealed that mRNA of neither IRS1 nor IRS2 showed any significant difference between lean controls, high IR-MO and T2D-MO subjects. Given that IRS1 is the main modulator of insulin signaling in adipose tissue [41], we found different levels of IRS1 protein expression in our three experimental groups. Of note, lean and high IR-MO groups had no differences in the IRS1 protein expression, fact that we have already reported in two populations with similar characteristics. However, visceral adipose tissue of low insulin resistant MO expresses high levels of IRS1 compared with lean and high IR-MO [25]. Thus, obesity might be associated with high levels of IRS1 protein expression whilst there is not insulin resistance. When IR occurs, IRS1 levels might decrease at levels comparable to those observed in lean, probably due to a feedback modulation induced by the inhibition of IRS1 activation (tyrosine phosphorylation) provoked by interleukins action. Moreover, we observed a decrease in the IRS1 protein expression in T2D-MO compared to high IR-MO and lean healthy donors, probably owing to the failure in B-cell function, decreasing the levels of insulin secretion.

Considered globally, the comparison of the VAT of these experimental groups has revealed that T2D-MO subjects exhibit the same pro-inflammatory profile than the high IR-MO subjects, characterized by elevated infiltration of M1 macrophages and high levels of IL-1β, IL-6, TNFα, JNK1/2, ERK1/2, STAT3 and NF-κB. Our work rule out the assumption that inflammation should be increased in obese people with T2D in comparison with high IR obese individuals. These findings indicate that other mechanisms, in addition to systemic and adipose tissue inflammation must be involved in the development of type 2 diabetes in obesity.

## Materials and Methods

### Subjects and Study Design

This study was undertaken in 40 morbidly obese (MO) subjects (body mass index (BMI) 56.10±1.31 kg/m2) (20 women and 20 men). As controls, 20 non-obese subjects (BMI 22.72±0.58 kg/m2) (6 women and 6 men) with no alterations in lipid or glucose metabolism, were selected. The weight of all individuals had been stable for at least one month and none had associated cardiovascular disease, arthritis, acute inflammatory disease, infectious disease or renal disease. None of the participants was receiving any antiadiabetic or other treatments that could alter the lipid profile or the metabolic parameters at the time of inclusion in the study. All patients signed informed consent forms and the study was approved by the ethics committees from Virgen de la Victoria Hospital, Malaga, Spain.

The morbidly obese patients were classified into two groups: patients having a HOMA-IR score>8 were considered high insulin resistant (high IR-MO) group and morbidly obese with type 2 diabetes mellitus (T2D-MO). Definition for T2D was based on fasting glucose concentration ≥126 mg/dl or glycemia ≥200 mg/dl 2 h after an OGTT. Clinical details of patients included in the study are indicated in Table I.

Visceral adipose tissue (VAT) biopsies were obtained from MO patients undergoing bariatric surgery using biliopancreatic diversion with the Scopinaro procedure or laparoscopic surgery procedures (hiatus hernia repair or cholecystectomies) in the case of the lean subjects. Tissue samples were washed in physiological saline, immediately frozen in liquid nitrogen and stored at –80°C for the different assays described below.

### Clinical Measurements

After overnight fast and inmediately before surgery, blood samples were obtained from the antecubital vein and placed in vacutainer tubes (BD vacutainerTM, London, UK). Serum was obtained by centrifugation for 10 min at 4000 rpm and immediately frozen at –20°C until late analysis. Levels of serum glucose, cholesterol, triglycerides and HDL cholesterol were analyzed using a Dimension autoanalyzer (Dade Behring Inc., Deerfield, IL) by enzymatic methods (Randox Laboratories Ltd., UK). LDL cholesterol was calculated from the Friedewald equation. Insulin was measured through radioimmunoassay supplied by BioSource International, Camarillo, S.A. Leptin and adiponectin were analyzed by enzyme immunoassay (ELISA) kits (DSL, Webster, TX, and DRG Diagnostics GmbH, Germany, respectively). The homeostasis model assessment of insulin resistance (HOMA-IR) was calculated following the equation described by Matthews et al., HOMA-IR = fasting insulin (mIU/mL)/fasting glucose (mmol/L)/22.5 [42].

### Visceral Adipose Tissue RNA Extraction

VAT RNA isolation started with the homogenization of the tissue using an ULTRATURRAX T25 basic (IKA Werke GmbH) and Trizol reagent (Invitrogen, Barcelona, Spain). Samples were purified using a RNAEasy Mini kit (QIAGEN, Barcelona, Spain) and treated with DNase (RNase-free DNase Set, Qiagen). The integrity of RNA was verified by optical density (OD) absorption ratio OD260/OD280 between 1.7 and 1.8.

### Reverse Transcription and Quantitative Real-time Polymerase Chain Reaction

For first strand cDNA synthesis, 1 µg of total RNA was reverse transcribed using random hexamers (Roche Diagnostic) as primers and Transcriptor Reverse Transcriptase (Roche Diagnostic). Gene expression was assessed by real time PCR using an ABI Prism 7000 Sequence Detection System (*Applied Biosystems, Darmstadt, Germany*), using TaqMan® technology suitable for relative genetic FASN expression quantification. The reaction was performed, following the manufacturers protocol, in a final volume of 25 µl. Commercially available and pre-validated TaqMan® primer/probe sets were used as follows:

Cyclophilin (*4333763, RefSeq. NM_002046.3, Cyclophilin A*), used as endogenous control for the target gene in each reaction, TNFα (*Hs00174128_m1, RefSeq. NM_000594.2, Tumor Necrosis Factor*), IL-6 (*Hs00174131_m1, RefSeq. NM_000600.2, Interleukin 6*), IL-1β (*Hs00174097_m1, RefSeq. NM_000576.2, Interleukin 1 beta*), IL-10 (Hs00961619_m1, RefSeq. NM_000572.2, *interleukin 10*), CD11b (*Hs01064804_m1, RefSeq. NM_000632.3, integrin, alpha M (complement component 3 receptor 3 subunit)),* PLAUR (*Hs00959822_m1, 2 RefSeq. NM_001005376.1, NM_002659.2, plasminogen activator, urokinase receptor*), MCP-1 (*Hs00234140_m1, RefSeq. NM_002982.3, chemokine (C-C motif) ligand 2*), CSF-3 (*Hs00357085_g1, 2 RefSeq. NM_172219.1, NM_000759.2, colony stimulating factor 3 (granulocyte)*), CD11c (Hs00174217_m1, RefSeq. NM_000887.3, integrin, alpha X), JNK1 (Hs00177083_m1, RefSeq. NM_002750.2, *mitogen-activated protein kinase 8*), IRS1 (H*s00178563_m1, RefSeq. NM_005544.2, Insulin receptor substrate 1*), IRS2 (*Hs00275843_s1, RefSeq, NM_003749.2, Insulin receptor substrate 2*), RELA (*Hs00153294_m1, RefSeq. NM_021975.2,* p65) and IκBα (*Hs00153283_m1, RefSeq. NM_020529.2, nuclear factor of kappa light polypeptide gene enhancer in B-cells inhibitor, alpha*).

The reactions consisted of an initial denaturing of 10 min at 95°C, then 40 cycles of 15 seconds denaturing phase at 95°C, and 1 minute annealing and extension phase at 60°C. A threshold cycle (Ct value) was obtained for each amplification curve and a ΔCt value was first calculated by subtracting the Ct value for human cyclophilin cDNA from the Ct value for each sample and transcript. Fold changes compared with the endogenous control were then determined by calculating 2-ΔCt. Every sample was performed in triplicate and negative controls were included in all the reactions. Test reproducibility for all investigated transcripts was less than 0.5% in intertest experiments and even lower in intratest experiments.

### Nuclear and Cytoplasmic Protein Isolation

Cytoplasmic and nuclear extracts were prepared from visceral adipose tissue using the NE-PER Nuclear and Cytoplasmic Extraction Reagents Kit (Pierce Chemical Co. Rockford, IL). 100 mg of tissue were homogenized in CER I Reagent with protease inhibitors (Sigma, St. Louis, MO), 100 mM phenylarsine oxide (PAO) (Sigma) and 1 mM sodium orthovanadate (Sigma) using an ULTRATURRAX T25 basic (IKA Werke GmbH, Staufen, Germany). After 10 min at 4°C, the CER II Reagent was added to the lysate. Samples were pelleted by centrifugation at 15000 g for 10 min at 4°C. The supernatants (cytoplasmic lysates) were recovered and frozen at −80°C.

The pellets (nuclear lysates) were incubated on ice for 40 min in NER Reagent with protease inhibitors (Sigma), 100 mM PAO (Sigma) and 1 mM sodium orthovanadate (Sigma). Samples were pelleted by centrifugation at 15000 g for 10 min at 4°C. Nuclear lysates were recovered and frozen at −80°C.

Reagent extraction volume was concentrated using Nanosep Centrifugal Devices (Pall Corporation, NY, USA). Protein concentrations were determined using BCA protein assay reagent (Pierce Chemical Co. Rockford) with bovine serum albumin as a standard. The fractionated proteins were assayed by Western blot and EMSA.

### Western Blot

25 µg of cytoplasmic cell lysates were separated by SDS-polyacrylamide gel electrophoresis (PAGE) (6–10%) and immunoblottings were incubated with the following antibodies: anti-phospho-JNK1/2, anti-IRS-1, anti-phospho STAT3 (Santa Cruz Biotechnology, CA, USA) and anti-phospho-ERK 1/2 (Cell Signaling Technology, Boston, USA). The inmunoblots were reprobed with human anti-JNK1/2, anti-β-actin, anti-STAT3 (Santa Cruz Biotechnology), and anti-ERK 1/2 (Calbiochem, La Jolla, CA).

### Electrophoretic Mobility Shift Assays (EMSA)

Nuclear extracts were tested for NF-κB binding activity, employing consensus oligonucleotides (50-AGTTGAGGGGACTTTCCCAGGC-30 and 30-TCAACTCCCCTGAAAGGGTCCG-50) (Santa Cruz Biotechnology), using the Digoxigenin EMSA kit from Roche Diagnostics (Basel, Switzerland). 25 µg nuclear protein was incubated with digoxigenin labelled NF-κB oligonucleotide in binding buffer containing 100 mM HEPES at pH 7.6, 5 mM Na2-EDTA, 50 mM (NH4)2SO4, 5 mM DTT, 1% (w/v) Tween 20, and 150 mM KCl, together with 1 µg of poly[d(I-C)], and poly L-lysine to a final volume of 20 µl. After 15 min of incubation at room temperature, the protein-DNA complexes were resolved on native 8% polyacrylamide gel in a 0.5X Tris-borate-EDTA buffer system and run at 200 V for 2 h. Gels were transferred to nylon membranes in a semidry transfer System (Bio-Rad) at 10 V and 300 mA for 30 min. The membranes were exposed to UV-light in a transilluminator for 5 min, and incubated with anti-digoxigenin alkaline phosphatase-conjugated antibody. Complexes were detected with CSPD chemiluminescent substrate (Roche Diagnostics) and exposed to Hyperfilm (GE Healthcare) in a film holder for 4–16 h at room temperature.

Antibody supershift assays were performed by incubation of the nuclear proteins with 4 µg of polyclonal affinity purified antibodies (Santa Cruz Biotechnology) against NF-B p50 (H-119), NF-κB p65 (C-20), and NF-κB p52 (K-27) subunits for 30 min on ice before adding the labeled probe. Specific competition control of unlabeled oligonucleotides at 125-fold excess was added to the binding reaction mixture.

### Immunohistochemical Analysis of Adipose Tissue

Visceral adipose tissue samples were fixed overnight at 4°C in 4% paraformaldehyde and embeded in paraffin. Section of 4 uM were dewaxed and rehydrated according to standard protocols. After washing slides with PBS 0.1M, pH 7.4 for 15 minutes, the sections were treated with PBS containing 10% methanol and 3% hydrogen peroxide to block endogenous peroxidase activity in dark during 20 min. Then, antigen retrieval procedures were performed (Target Retrieval Solution High pH, Dako, Denmark). Following several washes with PBS, they were exposed overnight to monoclonal mouse anti-human CD68 (Dako). Washed sections were then incubated with the appropiate biotinylated secondary antibodies for 1h (Polyclonal Rabbit Anti-Mouse immunoglobulins/Biotinylated (Dako) and Extravidin-peroxidase (Sigma) for 30 min. Staining was visualized using peroxidase substrate 3,3′-diaminobenzidine (Sigma). Sections were counten stained with Mayeŕs Hematoxylin (Sigma) and mounting solution and coverslips were added. Slides were observed under an Olympus BX41 microscope (Olympus, UK). Digital images were captured by a camera with an Olympus DP70 digital camera (Olympus, UK).

### IL-6 Determination

Plasma IL-6 levels were quantified using high-sensitivity enzyme-Linked immunosorbent assay (ELISA) (LINCO Research, St. Charles, MO), following the manufacturer’s instructions.

### Detection of CD11c Expression in ATMs by Flow Cytometry

Adipose tissue fractionation: Visceral adipose tissue from 8 morbidly obese patients (4 high IR-MO and 4 T2D-MO) was resuspended in digestion solution (5 ml/gr) (PBS, 1% BSA and 0.15% collagenase type I (Sigma)). The digestion was performed at 37°C using a shaker at 70 rpm for 70 min. After digestion, the samples were centrifuged at 500 g for 10 min at 24°C. The adipocyte fraction (floating) was eliminated and the solution containing the stroma vascular fraction was filtered using a 100 micras filter and then centrifuged at 400 g for 5 min at 24°C.

The stroma vascular fraction pellet was resuspended in 300 µl selection buffer (PBS, 2 mmol/L EDTA, and 0.5% BSA), and the CD34^−^ cells were selected using CD34 microbeads (Miltenyi Biotec S. L, Madrid, Spain) according to the manufacturer’s instructions. Thereafter, CD14^+^ cells were obtained using CD14 microbeads (Miltenyi).

Flow cytometry: Adipose tissue macrophage fraction (CD34−/CD14+) was incubated with 3% BSA solution for 10 min, followed by incubation with specific anti-human monoclonal antibody Cd11c-PE-Cy7 (eBioscience lnc, San Diego, CA, USA).

After washing, ATMs were analyzed using CyAnTM ADP High-speed Analyzer equipped with Summit 4.3 software (Beckman Coulter, Madrid, Spain). Nonspecific antibody conjugated to PE-Cy7 was used as negative control. The percentage of positive cells for CD11c antibody was determined by the percentage of cells exceeding the threshold obtained with nonspecific antibodies.

### Statistical Analysis

All data presented are given as means ± SEM. Statistical analyses were carried out with the statistical software package SPSS (version 15.0 for Windows; SPSS Iberica, Madrid, Spain). All the parameters studied were compared between male and female groups using a paired t-test. Then, the data were analyzed by analysis of variance one way (ANOVA). Post hoc statistical analysis was completed by using Duncan contrast statistic. Differences were considered statistically significant at p<0.05. The Spearman correlation coefficients were calculated to estimate the linear correlations between variables. The rejection level for a null hypothesis was p<0.05.
